# Transmitted drug resistance and molecular transmission network among treatment-naive HIV-1 patients in Wenzhou, China, 2020–2023

**DOI:** 10.1186/s12985-024-02528-2

**Published:** 2024-10-17

**Authors:** Tianran Zhang, Huifen Dou, Hui Ye, Han Tang, Weiqin Wang, Wenxue Hu, Binbin Lv, Mingshi Zhou, Hupiao Dai, Weilong Wang, Baochang Sun

**Affiliations:** 1 Wenzhou Municipal Center for Disease Control and Prevention, Wenzhou Municipal Institute of Health Supervision, No 41, Xin Cheng Road, Wenzhou, Wenzhou 325000 China; 2 Department of Infectious Diseases, Wenzhou Central Hospital Affiliated to Wenzhou, Wenzhou, 325000 China

**Keywords:** HIV-1, Transmitted drug resistance, Transmission cluster, Molecular epidemiology, Resistance mutations

## Abstract

**Background:**

Transmitted drug resistance (TDR) increases the risk of antiretroviral therapy (ART) failure in HIV-1 patients. This study investigated the molecular epidemiology of TDR and its transmission networks among newly diagnosed HIV-1 patients in Wenzhou, China.

**Methods:**

We enrolled 1878 ART-naive HIV-1 patients from January 2020 to October 2023. TDR was evaluated using the Stanford University HIV Drug Resistance Database. We performed phylogenetic analysis, genotyping, transmission clustering, and population-based TDR-related factor analysis.

**Results:**

Among 1782 patients with successful genotyping, TDR prevalence was 5.7%. Multivariable analysis identified CRF08_BC subtype (adjusted odds ratio [aOR] 18.59, 95% CI 3.79-336.18, *p* = 0.004), CD4 > 500 cells/mm³ (aOR 2.19, 95% CI 1.16–4.03, *p* = 0.013), and year 2023 (aOR 1.83, 95% CI 1.11–4.89, *p* = 0.039) as factors associated with higher TDR risk. The most prevalent NNRTI mutations were K103N, E138A, and V179E. Seven TDR transmission clusters were identified, notably one with V179D that expanded during 2020–2023.

**Conclusions:**

While TDR prevalence in Wenzhou remained lower than in other Chinese regions, an upward trend was observed. Most resistant individuals were in transmission clusters, predominantly middle-aged and elderly. NNRTI resistance was severe and concentrated in efavirenz, nevirapine, and rilpivirine. Enhanced HIV surveillance and wider free antiretroviral options are crucial to control drug-resistant HIV spread in Wenzhou.

**Supplementary Information:**

The online version contains supplementary material available at 10.1186/s12985-024-02528-2.

## Introduction

The human immunodeficiency virus (HIV) pandemic remains a significant global health challenge, affecting an estimated 39 million individuals worldwide [1]. In China, the HIV epidemic has escalated to over 1 million cases since the first reported case in 1985 [2]. The introduction of combination antiretroviral therapy (ART) in the mid-1990s dramatically improved outcomes for people living with HIV [3, 4], leading to the launch of China’s National Free Antiretroviral Treatment Program in 2003 [5].

However, the widespread use of ART has led to the emergence and spread of HIV drug resistance, posing new challenges. Drug resistance occurs when the virus accumulates mutations that reduce susceptibility to antiretroviral drugs [6]. In China, a meta-analysis estimated a 44.7% prevalence of acquired drug resistance among patients experiencing virologic failure [7].

Transmitted drug resistance (TDR), the transmission of drug-resistant HIV strains to treatment-naive individuals, is particularly concerning. TDR can compromise first-line ART efficacy [8], leading to earlier treatment failure and necessitating more expensive second-line regimens [9, 10]. The prevalence of TDR varies globally and has been increasing in many regions [11–13].

In China, a recent nationwide study found an overall TDR prevalence of 4.6% between 2001 and 2022, with an alarming 11.2% annual increase from 2016 to 2022 [14]. This trend underscores the urgent need for enhanced drug resistance monitoring and prevention efforts.

Wenzhou, with over 9 million inhabitants, faces a significant HIV burden. The estimated HIV prevalence is 0.2%, with more than 500 new cases reported annually since 2016. Heterosexual transmission accounts for over 60% of new infections [15]. The city’s unique characteristics, including high population mobility and a large number of overseas businessmen, contribute to the complex HIV epidemic [16–18].

Despite these challenges, research on TDR prevalence and transmission cluster analysis in Wenzhou remains scarce. To address this gap, we conducted a cross-sectional study to assess TDR epidemiology and characterize its transmission networks among newly diagnosed, ART-naive HIV-1 patients in Wenzhou from 2020 to 2023. By leveraging molecular epidemiology techniques, we aimed to provide comprehensive insights into the local TDR landscape, informing targeted prevention and treatment strategies.

## Methods

### Study design and participants

This cross-sectional study enrolled 1878 ART-naive HIV-1 patients newly diagnosed between January 1, 2020, and October 1, 2023, in Wenzhou. At enrollment, participants completed a questionnaire on demographics and HIV risk factors.

### Laboratory methods

RNA was extracted from isolated plasma (Qiagen, RNeasy Mini Kit) and amplified using nested PCR to obtain a 1197 bp target fragment spanning the full-length protease gene and the first 300 codons of the reverse transcriptase gene in the pol region (HXB 2: 2253–3450). The PCR protocol was briefly described as follows: the first round of amplification was performed using primers RT21_F (TGGAAATGTGGAAAAGAAGGAC) and RT21_R (CTGTATTTCAGCTATCAAGTCTTTTGATGGG) for reverse transcription, followed by a second round of amplification using primers PRO1_F (CAGAGCCAACAGCCCCACCA) and PRO1_R (CTGCCAATTCTAATTCTGCTTC). Subsequently, the PCR products were sent to Tsingke Biotechnology Co., Ltd for Sanger sequencing (ABI, 3730XL).

### Sequence analysis

The calibration and sequence splicing were conducted using Sequencer 4.10.1 (GeneCodes, Ann Arbor, MI). Additionally, the REGA HIV SUBTYPING TOOL V3.46 [19], COMET [20], and the HIV Gene Sequence Data Management and Analysis System in China [21] were employed to ascertain the subtypes. Uncommon circulating recombinant forms (CRFs) and unique recombinant forms (URFs) were identified using SimPlot++ [22]. For population-based TDR-related factor analysis, the Stanford University genotypic resistance interpretation algorithm (https://hivdb.stanford.edu/cpr/form/PRRT/) was employed to identify drug resistance mutations. According to the WHO 2021 HIV drug resistance report [23], we evaluated the resistance levels to 12 antiretroviral drugs, including efavirenz (EFV), nevirapine (NVP), abacavir (ABC), zidovudine (AZT), stavudine (D4T), didanosine (DDI), emtricitabine (FTC), lamivudine (3TC), tenofovir (TDF), atazanavir/ritonavir (ATV/r), darunavir/ritonavir (DRV/r), and lopinavir/ritonavir (LPV/r). Based on the Stanford University HIV Drug Resistance Database, the study population was subjected to analysis concerning resistance levels, which were categorized into Three levels for each ART drug: low-level resistance (LR, 15–29), intermediate resistance (IR, 30–59), and high-level resistance (HR, ≥ 60). The presence of one or more drugs with a resistance score of ≥ 15 was deemed to indicate resistance.

### Phylogenetic analysis and molecular networks

The sequences were aligned with reference sequences of the major subtypes using Clustal Omega [24]. The ModelFinder tool in IQ-TREE V 1.6.12 was employed to identify the optimal nucleotide substitution model: GTR + G + I [25]. A maximum likelihood phylogenetic tree was constructed using 1000 ultrafast bootstrap replicates [26] to test the confidence of the evolutionary tree branching. The hill-climbing nearest neighbor interchange function, which is included in the software, was employed to address the issue of model violation in ultrafast bootstrap. The constructed ML phylogenetic tree was imported into iTOL v5 [27] for visualization. The constructed tree was then imported into Clustal Picker [28] to identify potential transmission clusters, with the parameters genetic distance threshold set to 0.015 and node support threshold set to 0.95. The resulting visualization was exported to Cytoscape [29] for further analysis.

### Statistical analysis

A statistical analysis was conducted using the R language to organize the data obtained from the enrolled patients. The basic information of the participants was organized into categorical variables based on their values. Univariate and multivariate logistic regression analyses were performed to identify factors associated with TDR. Variables with a p-value of less than 0.1 in the univariate analysis were included in the multivariate regression analysis. A p-value of less than 0.05 was considered statistically significant in the multivariate regression analysis.

## Results

### Characteristics of the study population

**Fig. 1:**
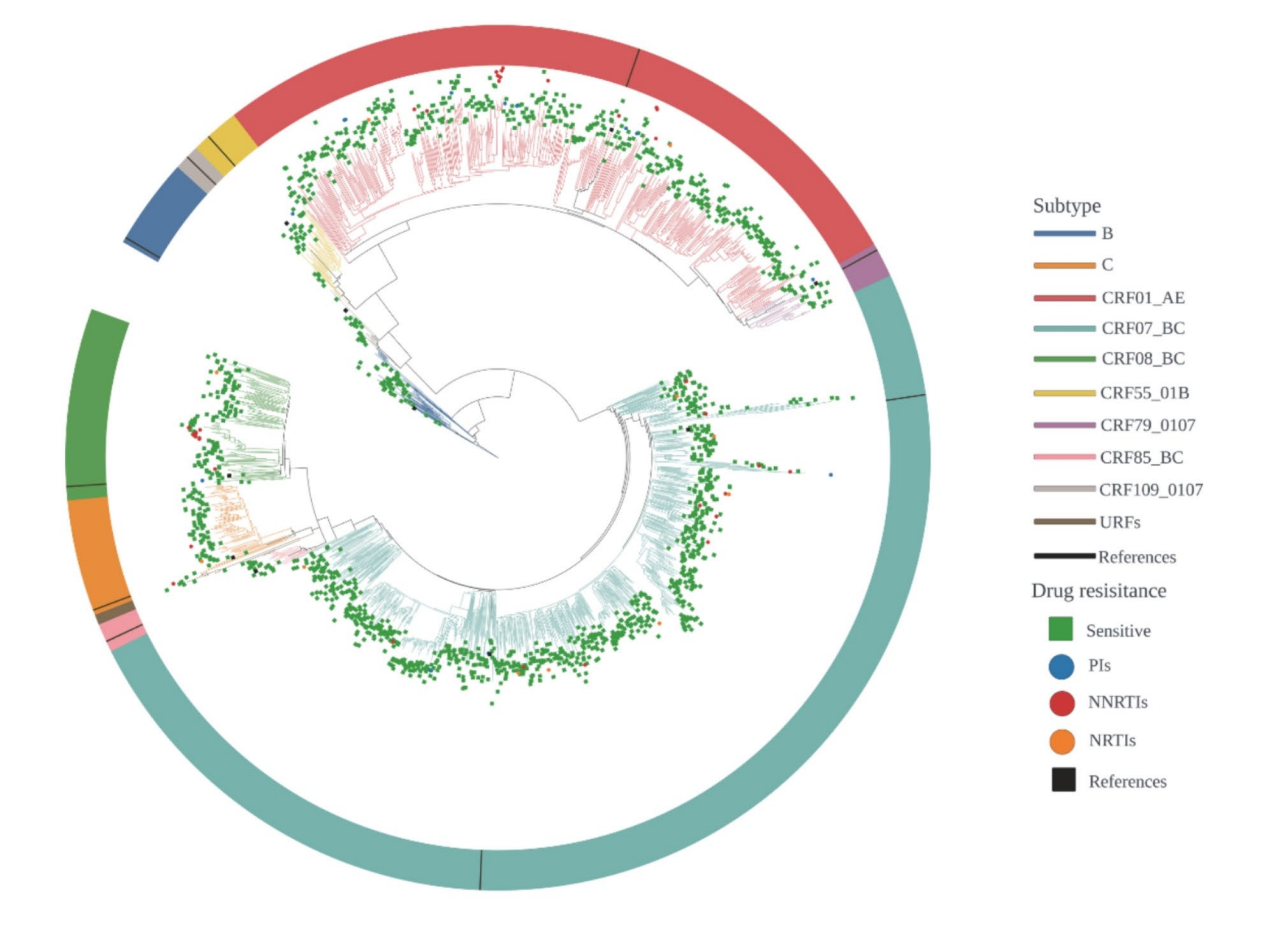
ML phylogenetic tree with 1,782 nucleotide sequences from newly diagnosed HIV patients

Between 2020 and 2023, we enrolled 1,878 study subjects and obtained 1,782 valid samples for sequencing analysis. We conducted statistical analysis on the demographic and social characteristics of these 1,782 patients (Table 1). The majority of the participants were male, accounting for 81.8% (1,458/1,782), with a median age of 46. The proportion of individuals over 50 was 42.5% (758/1,782), while those between 30 and 49 years old accounted for 37% (658/1,782). The main transmission route was heterosexual transmission, which accounted for 62.6% (1,116/1,782). 41.4% (738/1,782) of the patients were married, and 94.6% (1,686/1,782) were of Han ethnicity. 72.4% of the patients had an education level of either junior high school (35.4%, 631/1,783) or primary school and below (37%, 658/1,782). Among these 1,782 patients, the median CD4 cell count was 242. The main prevalent genotypes were CRF07_BC (52.1%, 928/1,782) and CRF01_AE (28.3%, 504/1,782), together accounting for 80.4% of the total (Fig. 1).

**Table 1:** Prevalence and determinants of transmitted drug resistance (TDR) among the participants

Variables	*N* (%)^a^	TDR (%)	X^2^	*P*-value	OR (95%CI)	*P*-value	Adjusted OR(95%CI)^b^	*P*-value
**Sex**								
Female	321(18.0%)	22(6.9%)	0.334	0.563	1			
Male	1461(82.0%)	79(5.4%)			0.777(0.485–1.295)	0.311		
**age**								
< 30	366(20.5%)	17(4.6%)	0.045	0.978	1			
30–49	660(37.0%)	35(5.3%)			1.150(0.644–2.130)	0.645		
≥ 50	756(42.5%)	49(6.5%)			1.423(0.824–2.576)	0.222		
**Marital status**								
Single	605(33.9%)	29(4.8%)	1.937	0.586	1			
Married	737(41.4%)	40(5.4%)			1.140(0.700-1.877)	0.601		
Divorced/widowed	414(23.2%)	28(6.8%)			1.445(0.843–2.471)	0.178		
Uknown	26(1.5%)	4(15.4%)			3.454(0.967–9.721)	0.031		
**Education**								
Primary	657(36.9%)	46(7%)	1.819	0.769	1			
Junior	630(35.3%)	37(4.3%)			0.829(0.527–1.294)	0.411		
Senior	249(14%)	9(3.6%)			0.498(0.225–0.986)	0.061		
College	242(13.6%)	9(3.7%)			0.513(0.232–1.016)	0.073		
Unknown	4(0.2%)	0			0	0		
**Ethnicity**								
Ethnic minorities	95(5.3%)	5(5.2%)	0.049	0.976	1			
Han	1686(94.6%)	96(5.7%)			1.087(0.476–3.138)	0.860		
Unknown	1(0.1%)	0			0	0		
**Transmission route**								
Heterosexual	1114(62.5%)	64(5.7%)	5.279	0.153	1			
Homosexual	618(34.7%)	31(5.0%)			0.866(0.551–1.335)	0.524		
Other	5(0.3%)	1(2.0%)			4.102(0.208–28.225)	0.210		
Unknown	45(2.5%)	5(11.1%)			2.051(0.690–4.931)	0.144		
**CD4 + T cell count(cells/mm3)**								
< 200	655(37.4%)	19(2.9%)	7.772	0.021	1			
200–499	907(51.9%)	28(3.1%)			0.860(0.543–1.369)	0.521	0.845(0.521–1.376)	0.495
≥ 500	188(10.7%)	13(6.9%)			2.003(1.098–3.554)	0.020	2.188(1.158–4.033)	0.013
**Subtypes**								
CRF01_AE	504(28.3%)	40(7.9%)			1			
CRF07_BC	928(52.1%)	25(2.7%)			0.320(0.190–0.531)	1.30E-05	1.401(0.284–25.369)	7.14E-06
CRF08_BC	131(7.3%)	31(23.7%)			3.588(2.131–6.006)	1.24E-06	18.585(3.792-336.183)	1.29E-06
other	219(12.3%)	5(2.3%)			0.270(0.092–0.634)	0.007	0	0.006
**Year**								
2020	521(29.2%)	31(5.9%)	6.680	0.083	1			
2021	512(28.7%)	21(4.1%)			0.676(0.378–1.186)	0.177	0.770(0.450–2.138)	0.386
2022	426(24.0%)	20(4.7%)			0.779(0.431–1.377)	0.396	0.947(0.701–3.207)	0.864
2023	323 (18.1%)	29(9.0%)			1.559(0.917–2.643)	0.098	1.834(1.114–4.893)	0.039

^a^ N: Number of Individuals ^b^ Adjusted for CD4 + T cell count, Year, Subtype

### Risk factors associated with TDR

The prevalence of TDR was 5.7% (101/1,782) in the total population, with an NNRTI resistance rate of 5.2% (92/1,782), an NRTI resistance rate of 0.4% (7/1,782), and a PI resistance rate of 0.1% (2/1,782). One patient had both NRTI and NNRTI resistance. Based on the results of univariate logistic regression analysis (Table 1), we selected three independent variables with p-values < 0.1 for subsequent multivariate logistic regression analysis: CD4 cell count, subtype, and year of infection. The multivariate logistic regression analysis revealed that CD4 cell count ≥ 500 (Adjusted OR(95%CI) = 2.188(1.158–4.033), p-value = 0.013), subtype, and year (2023, Adjusted OR(95%CI) = 1.834(1.114–4.893), p-value = 0.039) were significantly associated with the dependent variable (*p* ≤ 0.05).

Regarding the subtype, CRF01_AE was used as the reference group. Compared to CRF01_AE, CRF07_BC showed no significant difference in TDR prevalence (Adjusted OR(95%CI) = 1.401(0.284–25.369), p-value = 7.14E-06). However, CRF08_BC was associated with a significantly higher risk of TDR (Adjusted OR(95%CI) = 18.585(3.792-336.183), p-value = 1.29E-06).

Further analysis of epidemiological factors within each subtype revealed varying associations(Table 2). For CRF08_BC, which showed the highest TDR prevalence, marital status (χ²=2.379, *p* = 0.498) and transmission route (χ²=0.608, *p* = 0.738) were not significantly associated with TDR. However, the year of infection was significantly correlated with TDR (χ²=7.928, *p* = 0.048), with the highest TDR rate (31.0%) observed in 2020.

**Table 2:** Prevalence and determinants of transmitted Drug Resistance (TDR) stratified by HIV-1 subtypes

	CRF01_AE				CRF07_BC				CRF08_BC				Other			
Variables	*N*(%)	TDR (%)	X2	*P*-value	*N*(%)	TDR (%)	X2	*P*-value	*N*(%)	TDR (%)	X2	*P*-value	*N*(%)	TDR (%)	X2	*P*-value
**Sex**																
Female	90(17.9%)	6(6.7%)	0.077	0.782	141(15.2%)	4(2.8%)	4.80E-30	1	43(32.8%)	11(25.6%)	0.020	0.887	47(21.5%)	1(2.1%)	1.63E-28	1
Male	414(82.1%)	34(8.2%)			787(84.8%)	21(2.7%)			88(67.2%)	20(22.7%)			172(78.5%)	4(2.3%)		
**Age**																
< 30	134(26.6%)	6(4.5%)	5.844	0.054	178(19.2%)	4(2.2%)	0.301	0.860	17(13.0%)	5(29.4%)	0.371	0.831	37(16.9%)	1(2.7%	2.975	0.226
30–49	200(39.7%)	14(7.0%)			364(39.2%)	11(3.0%)			34(26.0%)	8(23.5%)			62(28.3%)	3(4.8%)		
≥ 50	170(33.7%)	20(11.8%)			386(41.6%)	10(2.6%)			80(61.1%)	18(22.5%)			120(54.8%)	1(0.8%)		
**Marital status**																
Single	204(40.5%)	8(3.9%)	12.692	0.005	309(33.3%)	8(2.6%	7.335	0.062	27(20.6%)	9(33.3%)	2.379	0.498	66(30.1%)	3(4.5%)	2.703	0.44
Married	180(35.7%)	14(7.8%)			388(41.8%)	10(2.6%)			64(48.9%)	15(23.4%)			105(48.0%)	2(1.9%)		
Divorced/widowed	116(23.0%)	17(14.7%)			217(23.4%)	5(2.3%)			36(27.5%)	6(16.7%)			44(20.1%)	0		
Uknown	5(1.0%)	1(20.0%)			14(1.5%)	2(14.3%)			4(3.1%)	1(25.0%)			4(1.8%)	0		
**Education**																
Primary	142(28.2%)	17(12.0%)	8.574	0.073	349(37.6%)	11(3.2%)	3.42	0.490	77(58.8%)	18(23.4%)	2.612	0.455	89(40.6%)	0	7.035	0.071
Junior	186(36.9%)	17(9.1%)			320(34.5%)	11(3.4%)			40(30.5%)	8(20.0%)			84(38.4%)	2(2.4%)		
Senior	86(17.1%)	3(3.5%)			132(14.2%)	2(1.5%)			8(6.1%)	2(25.0%)			24(11.0%)	1(4.2%)		
College	90(17.9%)	3(3.3%)			125(13.5%)	1(0.8%)			6(4.6%)	3(50.0%)			22(10.0%)	2(9.1%)		
Unknown	1(0.2%)	0			2(0.2%)	0			0	0			0	0		
**Ethnicity**																
Ethnic minorities	32(6.3%)	1(3.1%)	0.494	0.482	44(4.7%)	1(2.3%)	1.68E-27	1	10(7.6%)	3(30.0%)	0.011	0.918	9(4.1%)	0	0.245	0.885
Han	472(93.7%)	39(8.3%)			884(95.3%)	24(2.7%)			121(92.4%)	28(23.1%)			209(95.4%)	5(2.4%)		
Unknown	0	0			0	0							1	0		
**Transmission route**																
Heterosexual	300(59.5%)	20(6.7%)	12.826	0.005	565(60.9%)	15(2.7%)	5.095	0.165	113(86.3%)	28(24.8%)	0.608	0.738	136(62.1%)	2(1.5%)	2.838	0.417
Homosexual	192(38.1%)	18(9.4%)			343(37.0%)	8(2.3%)			13(9.9%)	2(15.4%)			70(32.0%)	2(2.9%)		
Other	1(0.2%)	1(100.0%)			2(0.2%)	0			5(3.8%)	1(20.0%)			2(0.9%)	0		
Unknown	11(2.2%)	1(9.1%)			18(1.9%)	2(11.1%)			0	0			11(5.0%)	1(9.1%)		
**CD4 + T cell count(cells/mm3)**																
< 200	225(44.6%)	18(8.0%)	2.539	0.281	336(36.2%)	12(3.6%)	3.355	0.187	37(28.2%)	8(21.6%)	1.932	0.381	90(41.1%)	1(1.1%)	4.559	0.102
200–499	226(44.8%)	15(6.6%)			498(53.7%)	9(1.8%)			78(59.5%)	17(21.8%)			105(47.9%)	2(1.9%		
≥ 500	53(10.5%)	7(13.2%)			94(10.1%)	4(4.3%)			16(12.2%)	6(37.5%)			24(11.0%)	2(8.3%)		
**Year**																
2020	147(29.2%)	9(6.1%)	7.234	0.065	252(27.2%)	3(1.2%)	11.04	0.012	58(44.3%)	18(31.0%)	7.928	0.048	64(29.2%)	1(1.6%)	2.215	0.529
2021	153(30.4%)	7(4.6%)			260(28.0%)	5(1.9%)			33(25.2%)	9(27.3%)			66(30.1%)	1(1.5%)		
2022	116(23.0%)	13(11.2%)			238(25.6%)	6(2.5%)			18(13.7%)	0			54(24.7%)	1(1.9%)		
2023	88(17.5%)	11(12.5%)			178(19.2%)	11(6.2%)			22(16.8%)	4(18.2%)			35(16.0%)	2(5.7%)		

In the CRF01_AE subtype, marital status (χ²=12.692, *p* = 0.005) and transmission route (χ²=12.826, *p* = 0.005) were significantly associated with TDR. Notably, divorced/widowed individuals (14.7%) and those reporting homosexual transmission (9.4%) had higher TDR rates.

For the CRF07_BC subtype, the year of infection was significantly associated with TDR (χ²=11.04, *p* = 0.012), with the highest TDR rate (6.2%) observed in 2023. Other factors such as gender, age, education level, ethnicity, and CD4 + T cell count were not significantly associated with TDR in this subtype.

### Analysis of resistance mutations and levels

**Fig. 2:**
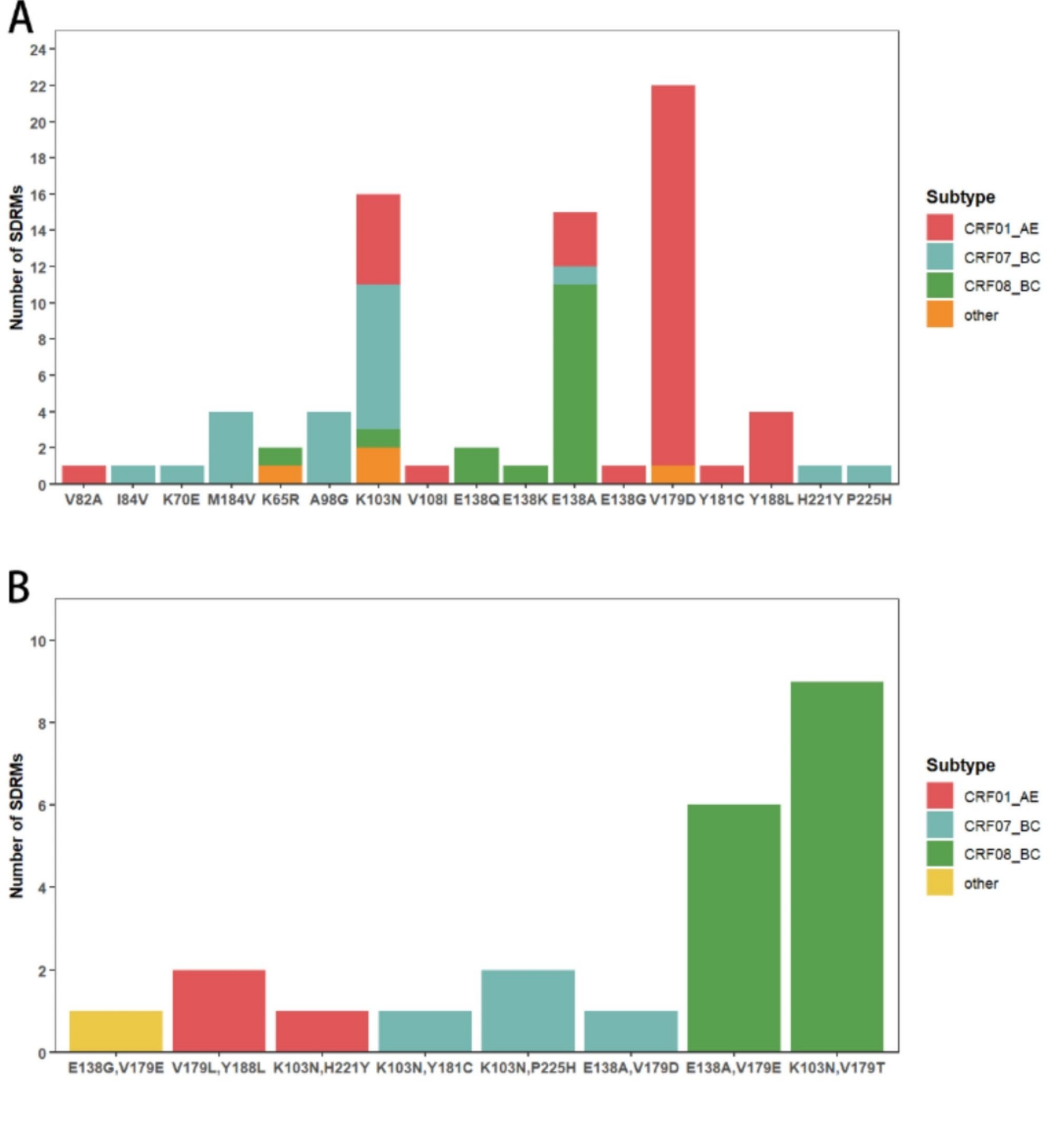
HIV-1 transmitted drug resistance mutations to PIs, NRTIs, and NNRTIs. (**a**) Single HIV-1 transmitted drug resistance mutations to PIs, NRTIs, and NNRTIs. (**b**) Multiple HIV-1 transmitted drug resistance mutations to PIs, NRTIs, and NNRTIs

In this study, we evaluated the resistance levels to 12 antiretroviral drugs according to the WHO 2021 HIV drug resistance report. NNRTI resistance was the most prevalent. The K103N mutation was detected in 15 patients (Table 3), with a predominant distribution in CRF07_BC (8 patients) and CRF01_AE (5 patients) subtypes, and additional cases in CRF08_BC (1 patient) and other subtypes (2 patients). The E138A mutation was identified in 17 patients, mainly in the CRF_08BC subtype, while the V179D mutation was detected in 22 patients, primarily in the CRF01_AE subtype (Fig. 2A). Notably, multiple patients were found to have more than one surveillance drug-resistance mutations (SDRMs), with the most common combination being E138A and V179E (Fig. 2B).

**Table 3:** Distribution of Surveillance Drug Resistance mutations (SDRMs) across HIV-1 subtypes

	CRF01_AE(*N*)	CRF07_BC(*N*)	CRF08_BC(*N*)	other(*N*)
**PIs**				
V82A	1			
I84V		1		
**NRTIs**				
K70E		1		
M184V		4		
K65R			1	1
**NNRTIs**				
A98G		4		
K103N	5	8	1	2
V108I	1			
E138G	1			
E138A	3	1	11	
E138Q			2	
E138K			1	
V179D	21			1
V179T				
V179E				
Y181C	1			
Y188L	4			
H221Y		1		
P225H		1		
K103N, H221Y	1			
V179L, Y188L	2			
K103N, Y181C		1		
K103N, P225H		2		
E138A, V179D		1		
E138A, V179E			6	1
K103N, V179T			9	

**Fig. 3:**
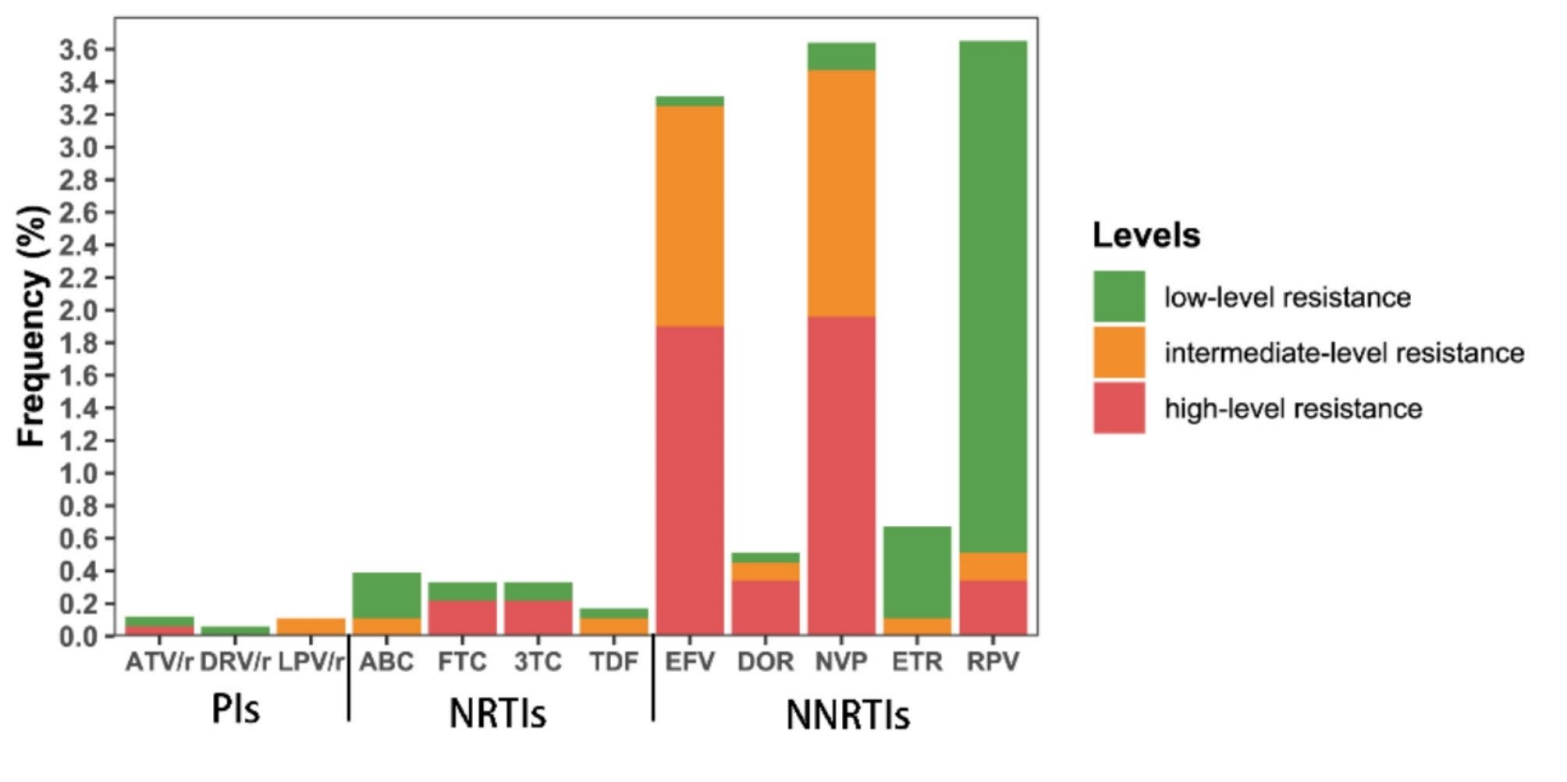
Frequency of Transmitted drug resistance in ART-naive HIV-1 patients between 2020 and 2023

The resistance levels associated with the identified mutations varied (Fig. 3). The K103N mutation clinically manifested as high-level resistance to the antiretroviral drugs EFV and NVP. The E138A mutation was associated with low-level resistance to RPV, while the V179D mutation corresponded to intermediate resistance to EFV, NVP, and RPV. Combining E138A and V179E led to intermediate resistance to EFV and RPV. NRTI resistance was detected in seven patients, four harboring the M184V mutation, clinically manifested as low-level resistance to ABC and high-level resistance to FTC and 3TC. These four patients were all of the CRF07BC subtype. PI resistance was relatively rare, detected in only two patients, with one exhibiting high-level resistance to ATV/r, low-level resistance to DRV/r, and intermediate resistance to LPV/r, and the other showing low-level resistance to ATV/r and intermediate resistance to LPV/r.

We found that PI and NRTI resistance were relatively uncommon in the population studied. In contrast, NNRTI resistance was the most severe, with a significant proportion of patients harboring mutations conferring varying resistance levels to different NNRTI drugs. The prevalence of resistance mutations and their associated resistance levels varied among different HIV-1 subtypes, with CRF07_BC, CRF01_AE, and CRF08_BC being the most affected.

### Characteristics of TDR Transmission clusters

**Fig. 4:**
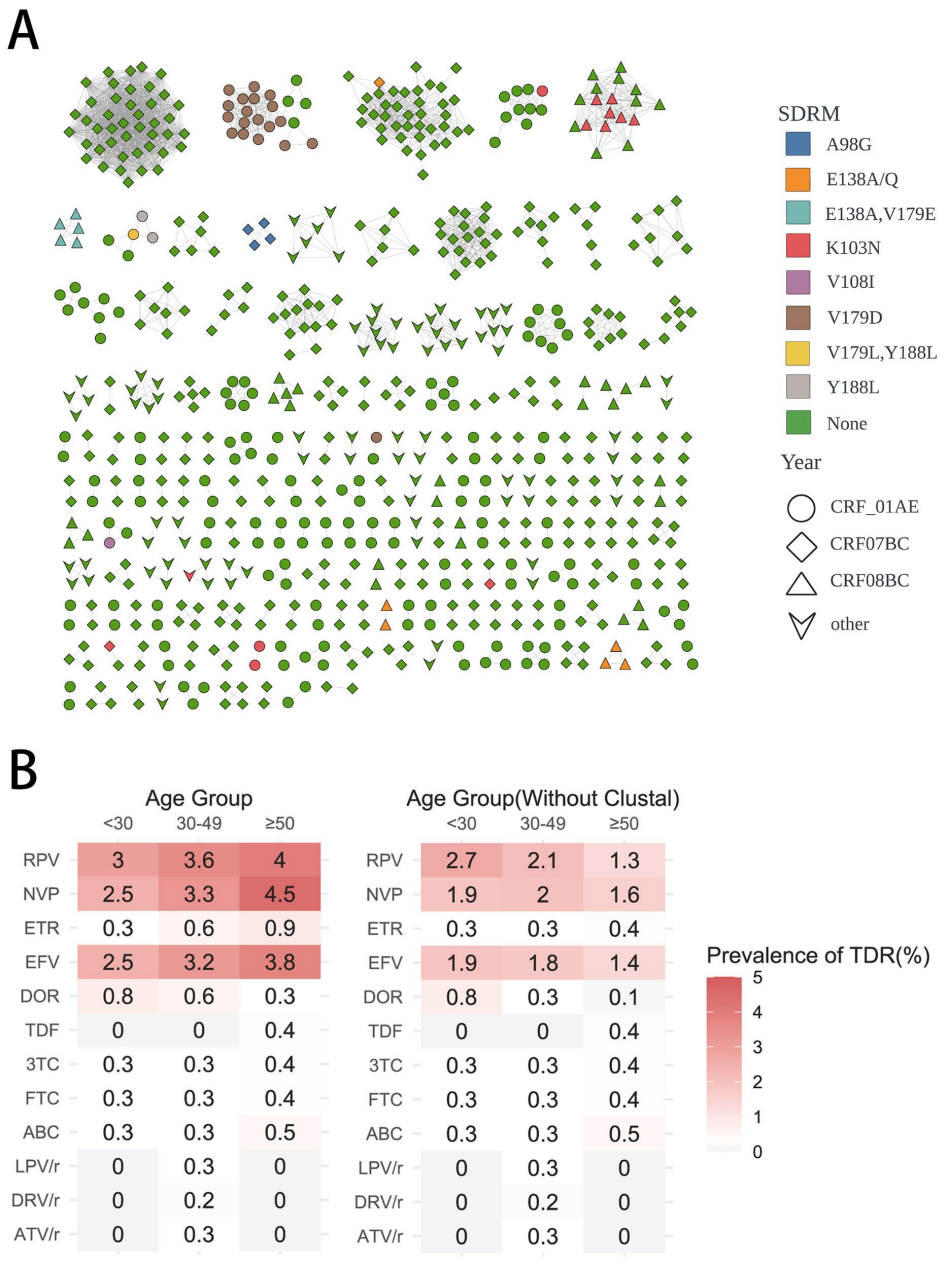
Drug resistance within HIV-1 transmission cluster. (**a**) Drug resistance within HIV-1 transmission clusters for different subtypes. (**b**) The study population was stratified into three age-based cohorts. The left column presents the prevalence of resistance to various ARVs among individuals within the TDR molecular transmission cluster. In contrast, the right column shows the prevalence of resistance to the same ARVs in individuals excluding those from the TDR molecular transmission cluster

We discovered seven transmission clusters containing TDR individuals (Fig. 4), all of which were NNRTI transmission clusters. The largest transmission cluster was cluster A, consisting of the CRF01_AE subtype with the V179D resistance mutation. Moreover, new resistant individuals continuously joined this transmission cluster from 2020 to 2023. Transmission clusters B and C were CRF07_BC and CRF01_AE transmission clusters, each containing only one resistant individual. The subtype of transmission cluster D was CRF08_BC, with the K103N mutation. However, unlike transmission cluster A, the resistant individuals in transmission cluster D were all newly diagnosed cases in 2020 and 2021. Transmission cluster E was also of the CRF08_BC subtype, with resistance mutations E138A and V179E, all newly diagnosed HIV cases in 2020 and 2021, with no new cases joining after 2021. The subtype of transmission cluster F was CRF01_AE, with the main resistance mutation being Y188L and one case having a combination of V179L and Y188L mutations. Although this transmission cluster had few resistant individuals, with only five individuals in the cluster, the resistant individuals were all newly diagnosed cases in 2022 and 2023. The subtype of transmission cluster G was CRF07_BC, with the A98G resistance mutation. Among the four resistant individuals included in this transmission cluster, three were newly diagnosed HIV cases in 2023. For the transmission clusters with resistant individuals appearing in the past four years, we found that their main transmission route was heterosexual. We found that, except for transmission cluster C, all the transmission clusters were primarily associated with heterosexual transmission and predominantly comprised middle-aged and elderly males (Supplementary Table 1).

Furthermore, when comparing the prevalence of transmitted drug resistance (TDR) between the study population that included molecular transmission clusters and the population without molecular transmission clusters, stratified by age groups, we observed that local molecular transmission clusters, indicative of aggregated transmission, had a more pronounced impact on the frequency of TDR in the middle-aged and older age groups compared to the younger age group (< 30 years old) (Fig. 4b). These findings may suggest that local aggregated transmission disproportionately affected middle-aged and older adults, with a lesser impact on individuals under 30 years of age.

## Discussion

This study represents the first comprehensive investigation of the prevalence of transmitted drug resistance in Wenzhou, China. We determined that the overall prevalence of TDR between 2020 and 2023 was 5.7%. The annual TDR prevalence in Wenzhou was 5.9%, 4.1%, 4.7%, and 9% in 2020, 2021, 2022, and 2023, respectively. Our findings revealed an overall TDR prevalence of 5.7%, which, although lower than pooled estimates from other regions in China during the same period [30–32], exhibited a concerning upward trend, increasing from 5.9% in 2020 to 9.0% in 2023.This underscores the urgent need for enhanced surveillance and prevention efforts to curtail the spread of drug-resistant HIV in this region.

Multivariable logistic regression analysis identified HIV-1 subtype, CD4 count, and year of diagnosis as significant predictors of TDR prevalence (*P* ≤ 0.05). Notably, HIV-1-infected individuals with CD4 counts greater than 500 cells/mm³ had a higher risk of developing TDR compared to those with lower CD4 counts. This finding is consistent with previous studies conducted in China [33], which have reported a similar association between higher CD4 counts and increased TDR prevalence. Furthermore, our analysis revealed that newly diagnosed HIV-1 patients in 2023 had a significantly higher risk of TDR compared to those diagnosed in other years, suggesting a concerning upward trend in HIV-1 TDR prevalence in Wenzhou in 2023. This rising TDR prevalence underscores the importance of strengthening surveillance efforts and adapting treatment guidelines to mitigate the impact of drug resistance on treatment outcomes.

The transmission cluster associated with the CRF01_AE subtype harbored the resistance mutations V179D, K103N, and Y188L, with V179D forming a large cluster of 18 resistant individuals (Fig. 4, cluster A). V179D, one of the most common surveillance drug-resistance mutations in this study, is also a prevalent NNRTI resistance mutation in China. The V179D mutation confers moderate resistance to EFV, NVP, and RPV. Recent studies have shown that V179D accounts for a higher proportion in Myanmar, Guangdong, Tianjin, and Shanghai in China, and is more frequently transmitted in the CRF01_AE-related subtype [34–37]. 

The CRF08_BC subtype demonstrated the highest risk of TDR, with a more than three-fold increased risk compared to CRF01_AE. The two transmission clusters associated with CRF08_BC harbored the resistance mutations K103N (Fig. 4, cluster D) and E138A/V179E (Fig. 4, cluster E). K103N, a prevalent NNRTI resistance mutation worldwide, confers high-level resistance to EFV and NVP, and its proportion among resistant strains is relatively high in Brazil, Spain, and sub-Saharan Africa [38–40]. Reports of resistance sites simultaneously containing E138A and V179E are rare. We found only one study from Scotland reported an outbreak involving both E138A and V179E resistance sites [41]. E138A, a polymorphic accessory mutation, confers low-level resistance to RPV, while V179E, a non-polymorphic mutation, is associated with resistance to ETR and RPV.

Unlike other densely populated cities in northern Zhejiang, such as Ningbo and Hangzhou [33, 42], the main route of HIV transmission in Wenzhou is through heterosexual contact. In this study, we found that six out of the seven identified drug-resistant transmission clusters were primarily associated with heterosexual transmission. Further analysis revealed that these heterosexual transmission clusters were a significant contributing factor to the prevalence of TDR among middle-aged and elderly populations in Wenzhou. This finding suggests that promiscuous sexual behavior among middle-aged and elderly individuals may be a key driver of clustered TDR transmission in the Wenzhou area.

The highly concentrated distribution of HIV-1 transmitted drug resistance (TDR) sites in Wenzhou, compared to other regions, is a significant finding of this study [43]. Among the 101 HIV-1 patients with TDR, 79.2% (80/101) harbored one or more of the K103N, E138A, and V179D mutations, which confer resistance to non-nucleoside reverse-transcriptase inhibitors (NNRTIs). Moreover, 92.1% (93/101) of TDR patients resisted at least one of the three NNRTIs: EFV, NVP, and RPV. This concentrated resistance pattern in the Wenzhou area may be attributed to two main factors.

First, despite the Chinese guidelines for acquired immunodeficiency syndrome (AIDS) diagnosis and treatment recommending a regimen of two NRTIs plus one integrase inhibitor (LPV/r or DRV/r) since 2018, patients often opt for free NNRTI drugs, with EFV and NVP being the most commonly used, followed by RPV. This preference is likely due to the exclusion of LPV/r and DRV/r from the list of drugs covered by the national free antiretroviral treatment policy. Consequently, the TDR prevalence of EFV and NVP has shown an increasing trend from 2021 to 2023 (Supplementary Table 2), consistent with findings from other regions in China [14]. Although RPV has a lower moderate-to-high resistance level than EFV and NVP, the proportion of low-level resistance is higher, and the upward trend in 2023 is evident. To address this issue, we recommend that the government expand the range of free drug choices and introduce policies to provide free resistance testing for patients before treatment to better guide subsequent medication use.

Second, the concentrated resistance pattern in the Wenzhou area may be related to the clustered transmission of HIV-1. Our transmission cluster analysis revealed that the two SDRMs, K103N, and V179D, have corresponding large resistant transmission clusters (cluster A and cluster D), while E138A is included in transmission cluster E. Furthermore, the main transmission route in Wenzhou is sexual transmission, and the hidden nature of this transmission route makes prevention and intervention more challenging, leading to the clustered transmission of resistance in the Wenzhou area. Our investigation also revealed a large population of people who leave Wenzhou for business and may engage in high-risk sexual behaviors upon returning, contributing to the epidemic spread of HIV. Therefore, increased attention to key populations is essential to curb the spread of resistant strains.

This study has several limitations due to various factors. For instance, the time of infection and the time of diagnosis for newly diagnosed patients may not be consistent. Some patients might not have been aware of their infection risk until they had been infected for a long time at the time of diagnosis. This discrepancy between the actual time of infection and the time of diagnosis could potentially lead to an underestimation of the prevalence of transmitted drug resistance in the study population, as some individuals with long-standing infections may have been misclassified as recently infected based on their clinical presentation or CD4 count at the time of diagnosis.

## Conclusions

In conclusion, this comprehensive study of 1,782 ART-naïve HIV-1 patients in Wenzhou, China, from 2020 to 2023 revealed a diverse and complex distribution of HIV-1 genotypes and mutation sites, with CRF07_BC and CRF01_AE being the dominant subtypes. The overall prevalence of TDR remained at a low level over the 4-year study period. However, more than half of the TDR patients were included in transmission clusters. This finding highlights the need for continued efforts in HIV drug resistance management in Wenzhou. Specifically, there is a need to strengthen prevention strategies, enhance monitoring systems, and implement targeted interventions to reduce both the prevalence and transmission of TDR.Targeted interventions addressing the unique transmission dynamics in this region, particularly among middle-aged and older heterosexual individuals, are crucial for the effective control of HIV drug resistance.

## Electronic supplementary material

Below is the link to the electronic supplementary material.

Supplementary Material 1

## Data Availability

No datasets were generated or analysed during the current study.

## Abbreviations

TDR: Transmitted drug resistance
NNRTIs: Nonnucleoside reverse transcriptase inhibitors
NRTIs: Nucleoside reverse transcriptase inhibitors
PIs: Protease inhibitors
ART: Antiretroviral therapy
AIDS: Acquired immunodeficiency syndrome
HIV: Human immunodeficiency viruses
SDRMs: Surveillance/transmitted drug resistance mutations
URFs: Unique recombinant forms
CRFs: Circulating recombinant forms
