# Supplementary material for: Transmitted drug resistance and molecular transmission network among treatment-naive HIV-1 patients in Wenzhou, China, 2020–2023
Source: Virol J. 2024 Oct 17;21:257. doi: 10.1186/s12985-024-02528-2 (PMC11487810; doi:10.1186/s12985-024-02528-2)
Supplement: Supplementary file 1 — Supplementary Material 1 [file 12985_2024_2528_MOESM1_ESM.docx]

Supplementary Table 1

| Clustal name | subtype | age | SDRM | Transmission route | year |
| --- | --- | --- | --- | --- | --- |
| Clustal_A | CRF01_AE | 73 | V179D | Heterosexual | 2020 |
| Clustal_A | CRF01_AE | 74 | V179D | Heterosexual | 2020 |
| Clustal_A | CRF01_AE | 75 | V179D | Heterosexual | 2020 |
| Clustal_A | CRF01_AE | 76 | V179D | Heterosexual | 2020 |
| Clustal_A | CRF01_AE | 76 | V179D | Heterosexual | 2020 |
| Clustal_A | CRF01_AE | 41 | V179D | Heterosexual | 2021 |
| Clustal_A | CRF01_AE | 46 | V179D | Homosexual | 2021 |
| Clustal_A | CRF01_AE | 48 | None | Homosexual | 2021 |
| Clustal_A | CRF01_AE | 74 | V179D | Heterosexual | 2021 |
| Clustal_A | CRF01_AE | 79 | V179D | Heterosexual | 2021 |
| Clustal_A | CRF01_AE | 29 | V179D | Homosexual | 2022 |
| Clustal_A | CRF01_AE | 34 | V179D | Homosexual | 2022 |
| Clustal_A | CRF01_AE | 45 | V179D | Homosexual | 2022 |
| Clustal_A | CRF01_AE | 46 | None | Heterosexual | 2022 |
| Clustal_A | CRF01_AE | 46 | None | Unknown | 2022 |
| Clustal_A | CRF01_AE | 47 | V179D | Heterosexual | 2022 |
| Clustal_A | CRF01_AE | 67 | V179D | Homosexual | 2022 |
| Clustal_A | CRF01_AE | 73 | V179D | Heterosexual | 2022 |
| Clustal_A | CRF01_AE | 32 | None | Homosexual | 2023 |
| Clustal_A | CRF01_AE | 48 | V179D | Heterosexual | 2023 |
| Clustal_A | CRF01_AE | 59 | V179D | Heterosexual | 2023 |
| Clustal_A | CRF01_AE | 59 | V179D | Heterosexual | 2023 |
| Clustal_B | CRF07_BC | 42 | None | Heterosexual | 2020 |
| Clustal_B | CRF07_BC | 44 | None | Homosexual | 2020 |
| Clustal_B | CRF07_BC | 47 | None | Heterosexual | 2020 |
| Clustal_B | CRF07_BC | 48 | None | Heterosexual | 2020 |
| Clustal_B | CRF07_BC | 48 | None | Heterosexual | 2020 |
| Clustal_B | CRF07_BC | 56 | None | Heterosexual | 2020 |
| Clustal_B | CRF07_BC | 57 | None | Heterosexual | 2020 |
| Clustal_B | CRF07_BC | 61 | None | Heterosexual | 2020 |
| Clustal_B | CRF07_BC | 61 | None | Heterosexual | 2020 |
| Clustal_B | CRF07_BC | 62 | None | Heterosexual | 2020 |
| Clustal_B | CRF07_BC | 64 | None | Heterosexual | 2020 |
| Clustal_B | CRF07_BC | 67 | None | Heterosexual | 2020 |
| Clustal_B | CRF07_BC | 71 | None | Heterosexual | 2020 |
| Clustal_B | CRF07_BC | 73 | None | Heterosexual | 2020 |
| Clustal_B | CRF07_BC | 77 | None | Heterosexual | 2020 |
| Clustal_B | CRF07_BC | 22 | None | Heterosexual | 2021 |
| Clustal_B | CRF07_BC | 31 | None | Heterosexual | 2021 |
| Clustal_B | CRF07_BC | 34 | None | Heterosexual | 2021 |
| Clustal_B | CRF07_BC | 41 | None | Heterosexual | 2021 |
| Clustal_B | CRF07_BC | 49 | E138A | Heterosexual | 2021 |
| Clustal_B | CRF07_BC | 49 | None | Heterosexual | 2021 |
| Clustal_B | CRF07_BC | 51 | None | Heterosexual | 2021 |
| Clustal_B | CRF07_BC | 53 | None | Heterosexual | 2021 |
| Clustal_B | CRF07_BC | 56 | None | Heterosexual | 2021 |
| Clustal_B | CRF07_BC | 57 | None | Heterosexual | 2021 |
| Clustal_B | CRF07_BC | 62 | None | Heterosexual | 2021 |
| Clustal_B | CRF07_BC | 66 | None | Heterosexual | 2021 |
| Clustal_B | CRF07_BC | 40 | None | Heterosexual | 2022 |
| Clustal_B | CRF07_BC | 46 | None | Heterosexual | 2022 |
| Clustal_B | CRF07_BC | 63 | None | Heterosexual | 2022 |
| Clustal_B | CRF07_BC | 64 | None | Heterosexual | 2022 |
| Clustal_B | CRF07_BC | 64 | None | Homosexual | 2022 |
| Clustal_B | CRF07_BC | 23 | None | Heterosexual | 2023 |
| Clustal_B | CRF07_BC | 47 | None | Heterosexual | 2023 |
| Clustal_B | CRF07_BC | 57 | None | Heterosexual | 2023 |
| Clustal_B | CRF07_BC | 58 | None | Heterosexual | 2023 |
| Clustal_B | CRF07_BC | 58 | None | Heterosexual | 2023 |
| Clustal_B | CRF07_BC | 61 | None | Heterosexual | 2023 |
| Clustal_B | CRF07_BC | 71 | None | Heterosexual | 2023 |
| Clustal_C | CRF01_AE | 22 | None | Homosexual | 2020 |
| Clustal_C | CRF01_AE | 23 | None | Homosexual | 2020 |
| Clustal_C | CRF01_AE | 23 | None | Homosexual | 2020 |
| Clustal_C | CRF01_AE | 28 | None | Homosexual | 2020 |
| Clustal_C | CRF01_AE | 29 | None | Homosexual | 2020 |
| Clustal_C | CRF01_AE | 23 | None | Homosexual | 2021 |
| Clustal_C | CRF01_AE | 74 | None | Heterosexual | 2021 |
| Clustal_C | CRF01_AE | 59 | None | Heterosexual | 2022 |
| Clustal_C | CRF01_AE | 22 | None | Homosexual | 2023 |
| Clustal_C | CRF01_AE | 24 | K103N | Homosexual | 2023 |
| Clustal_D | CRF08_BC | 54 | None | Heterosexual | 2020 |
| Clustal_D | CRF08_BC | 54 | K103N | Heterosexual | 2020 |
| Clustal_D | CRF08_BC | 55 | K103N | Heterosexual | 2020 |
| Clustal_D | CRF08_BC | 55 | None | Heterosexual | 2020 |
| Clustal_D | CRF08_BC | 70 | K103N | Heterosexual | 2020 |
| Clustal_D | CRF08_BC | 74 | None | Heterosexual | 2020 |
| Clustal_D | CRF08_BC | 74 | None | Heterosexual | 2020 |
| Clustal_D | CRF08_BC | 78 | K103N | Heterosexual | 2020 |
| Clustal_D | CRF08_BC | 82 | K103N | Heterosexual | 2020 |
| Clustal_D | CRF08_BC | 85 | K103N | Heterosexual | 2020 |
| Clustal_D | CRF08_BC | 25 | None | Homosexual | 2021 |
| Clustal_D | CRF08_BC | 31 | K103N | Heterosexual | 2021 |
| Clustal_D | CRF08_BC | 53 | None | Homosexual | 2021 |
| Clustal_D | CRF08_BC | 57 | None | Heterosexual | 2021 |
| Clustal_D | CRF08_BC | 62 | None | Heterosexual | 2021 |
| Clustal_D | CRF08_BC | 58 | None | Heterosexual | 2023 |
| Clustal_D | CRF08_BC | 67 | None | Heterosexual | 2023 |
| Clustal_D | CRF08_BC | 70 | None | Heterosexual | 2023 |
| Clustal_E | CRF08_BC | 63 | None | Heterosexual | 2020 |
| Clustal_E | CRF08_BC | 31 | None | Heterosexual | 2021 |
| Clustal_E | CRF08_BC | 61 | None | Heterosexual | 2021 |
| Clustal_E | CRF08_BC | 67 | None | Heterosexual | 2021 |
| Clustal_E | CRF08_BC | 79 | None | Unknown | 2021 |
| Clustal_F | CRF01_AE | 32 | None | Homosexual | 2021 |
| Clustal_F | CRF01_AE | 51 | None | Heterosexual | 2021 |
| Clustal_F | CRF01_AE | 72 | Y188L | Heterosexual | 2022 |
| Clustal_F | CRF01_AE | 32 | V179L,Y188L | Homosexual | 2023 |
| Clustal_F | CRF01_AE | 49 | Y188L | Homosexual | 2023 |
| Clustal_G | CRF07_BC | 53 | A98G | Heterosexual | 2022 |
| Clustal_G | CRF07_BC | 60 | A98G | Heterosexual | 2023 |
| Clustal_G | CRF07_BC | 70 | A98G | Heterosexual | 2023 |
| Clustal_G | CRF07_BC | 70 | A98G | Unknown | 2023 |

Supplementary Table 2

|  | 2020 | 2021 | 2022 | 2023 |
| --- | --- | --- | --- | --- |
| **PI** | | | | |
| ATV/r | 0.19 | 0.00 | 0.00 | 0.31 |
| DRV/r | 0.19 | 0.00 | 0.00 | 0.00 |
| LPV/r | 0.19 | 0.00 | 0.00 | 0.31 |
| **NTRI** | | | | |
| ABC | 0.19 | 0.20 | 0.70 | 0.62 |
| FTC | 0.00 | 0.20 | 0.70 | 0.62 |
| 3TC | 0.00 | 0.20 | 0.70 | 0.62 |
| TDF | 0.19 | 0.20 | 0.00 | 0.31 |
| **NNTRI** | | | | |
| DOR | 0.00 | 0.00 | 0.70 | 1.86 |
| EFV | 3.26 | 1.95 | 3.52 | 5.26 |
| ETR | 0.19 | 0.98 | 0.00 | 1.86 |
| NVP | 3.07 | 2.34 | 3.76 | 6.19 |
| RPV | 1.92 | 1.56 | 1.23 | 2.17 |
